# Extended-Duration High-Voltage Pulsed Current Stimulation for Persistent Lateral Ankle Pain With Local Hypersensitivity After Talonavicular Coalition Resection: A Case Report

**DOI:** 10.7759/cureus.113422

**Published:** 2026-07-26

**Authors:** Aya Okamichi, Haruka Ikenaga, Haruka Kawaguchi, Ayane Kubo

**Affiliations:** 1 Department of Physical Therapy, School of Health Sciences at Narita, International University of Health and Welfare, Chiba, JPN; 2 Department of Rehabilitation Medicine, J Medical Oyumino, Chiba, JPN; 3 Rehabilitation Center, Kikkoman General Hospital, Noda, JPN; 4 Department of Rehabilitation Medicine, Kamisu Saiseikai Hospital, Kamisu, JPN

**Keywords:** ankle instability, electrical stimulation, high-voltage pulsed current, lateral ankle pain, pain hypersensitivity, persistent postoperative pain, pressure pain threshold, sinus tarsi, talonavicular coalition, tarsal coalition

## Abstract

Persistent lateral ankle pain after talonavicular coalition resection can be difficult to manage when mechanical factors alone do not explain the symptoms. This case highlights the clinical relevance of reassessing localized mechanical pain hypersensitivity at the sinus tarsi and adjusting electrical stimulation parameters based on the patient’s clinical response.

We describe a 22-year-old woman with persistent right lateral ankle pain during gait 14 months after resection of a talonavicular coalition. Analgesic medication had provided insufficient relief, and medication use remained unchanged throughout the treatment period. Initial assessment showed tenderness around the anterior talofibular and calcaneofibular ligaments, positive anterior drawer and inversion stress tests, restricted ankle range of motion, early heel-off during the right stance phase, and increased hip and knee flexion during gait. Walking pain was 5/10 on the numerical rating scale (NRS). Taping was applied for four weeks to reduce mechanical stress associated with lateral instability, but walking pain persisted at NRS 5/10. Reassessment identified the sinus tarsi as the primary pain site, with tactile hypersensitivity and a markedly reduced pressure pain threshold (PPT) on the affected side.

High-voltage pulsed current (HVPC) stimulation was applied to the sinus tarsi for 10 minutes per session, four to five times weekly for five weeks. Pain transiently decreased to NRS 4/10 immediately after sessions, but sustained improvement was not achieved, and walking pain subsequently worsened to NRS 6/10. The stimulation duration was then extended to 30 minutes per session while the same electrode placement and intensity range were maintained. After this sequential change, walking pain decreased to NRS 2/10, and the affected-side PPT, gait findings, and affected forefoot loading improved. At the four-week follow-up, walking pain remained at NRS 0-1/10, with no adverse events.

This case suggests that localized mechanical pain hypersensitivity at the sinus tarsi should be considered when persistent lateral ankle pain after talonavicular coalition resection cannot be explained by mechanical factors alone. The clinical improvement observed after extending the stimulation duration represents a temporal association and should be interpreted as a hypothesis-generating observation. Because this was a sequential single-case design without a control condition, washout period, or repeated baseline, the improvement cannot be attributed solely to the longer stimulation duration. Alternative explanations, including cumulative treatment effects, natural recovery, placebo response, and regression to the mean, cannot be excluded.

## Introduction

Tarsal coalition is an abnormal osseous, cartilaginous, or fibrous union between two or more tarsal bones and is generally considered a congenital or developmental anomaly [1]. This abnormal union can restrict foot motion and increase mechanical load on adjacent joints and soft tissues, resulting in pain, restricted range of motion, recurrent ankle sprains, and gait disturbance [1]. Conservative treatment is usually the first-line approach for symptomatic tarsal coalition, but surgical resection may be considered when pain or functional impairment persists despite conservative management [2]. Talonavicular coalition is a relatively rare type of tarsal coalition [3], and reports of long-standing postoperative pain following its resection remain limited.

Although clinical outcomes after coalition resection are generally favorable, identifying the cause of persistent postoperative pain and determining an appropriate rehabilitation strategy can be challenging [2]. Lateral ankle pain is often attributed to mechanical factors, including lateral ligament instability, stress on tissues surrounding the sinus tarsi, impingement, and soft-tissue injury [4]. The sinus tarsi is an anatomically complex space between the talus and calcaneus that contains ligamentous, vascular, adipose, and neural structures and is clinically relevant in patients with lateral hindfoot pain and instability [5,6]. Therefore, when mechanical control alone does not achieve adequate improvement, localized mechanical pain hypersensitivity at the sinus tarsi should also be considered.

High-voltage pulsed current (HVPC) stimulation is a physical modality used in rehabilitation for pain relief and edema reduction, among other purposes [7]. The analgesic effects of electrical stimulation are thought to involve mechanisms such as segmental inhibition of nociceptive input and activation of descending pain-inhibitory pathways, and these effects may be influenced by stimulation parameters, including intensity, frequency, pulse width, electrode placement, and stimulation duration [8]. However, evidence regarding how adjustment of stimulation duration may influence clinical responses in patients with persistent postoperative lateral ankle pain is limited.

The purpose of this report is to describe the clinical course of a patient with lateral ankle pain persisting for more than one year after talonavicular coalition resection, in whom both mechanical factors and localized mechanical pain hypersensitivity at the sinus tarsi were considered based on clinical findings. We also describe the clinical changes observed after sequential extension of HVPC stimulation duration, while recognizing that this single-case design cannot establish a causal relationship.

## Case presentation

Patient background

The patient was a 22-year-old woman with a height of 160 cm, a weight of 58.1 kg, and a body mass index of 22.7 kg/m². She was referred to an orthopedic surgeon after restricted right ankle range of motion was noted and was diagnosed with right talonavicular coalition. She subsequently underwent resection of the coalition. After surgery, she ambulated with non-weight-bearing followed by partial weight-bearing for a certain postoperative period; however, the exact duration could not be confirmed from the available records. No specialized postoperative rehabilitation program was performed. Despite 14 months elapsing since surgery, right lateral ankle pain during gait persisted, and she presented for physical therapy evaluation with the chief complaint of “pain when walking.” Her history included right ankle inversion sprain. She was independent in activities of daily living and did not participate in regular sports or special exercise activities. Her activity level did not substantially change during the observation period. She had been taking analgesic medication but had not achieved adequate pain relief; the type, dose, and frequency of medication remained unchanged throughout the observation period. She reported no known family history of tarsal coalition or similar foot disorders.

Initial assessment

**Figure 1:**
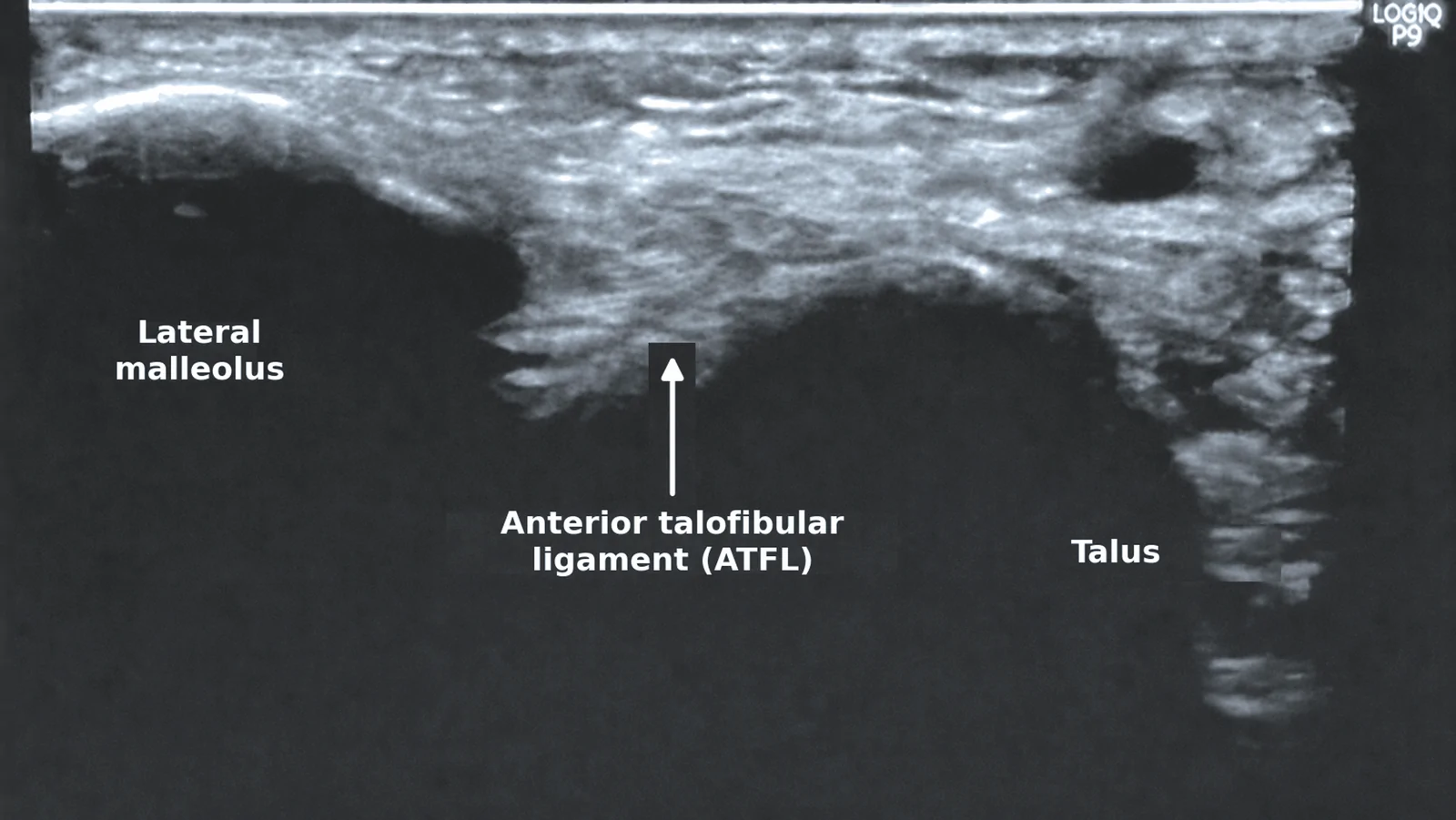
Ultrasonographic findings at initial assessment Static ultrasound image of the lateral ankle at initial assessment. The anterior talofibular ligament (ATFL) region is indicated by the arrow; its continuity was indistinct, a finding suggestive of lateral ligament injury. The lateral malleolus and talus are labeled for anatomical orientation. Patient identifiers were removed.

Initial assessment revealed tenderness around the anterior talofibular ligament, calcaneofibular ligament, calcaneocuboid joint, and navicular bone. Lateral ankle pain occurred during weight-bearing and push-off, and supination produced stretching pain around the calcaneofibular ligament. The anterior drawer and inversion stress tests were positive. Ankle range of motion was 23°/35° (right/left) for dorsiflexion with the knee flexed, 15°/27° for dorsiflexion with the knee extended, and 35°/60° for plantarflexion, indicating restricted motion on the affected side. Ultrasonography showed indistinct continuity of the anterior talofibular ligament, a finding suggestive of lateral ligament injury (Figure 1). Indistinct continuity of the calcaneofibular ligament was also noted in a different scan plane; however, this finding was not captured in a saved image or video. The sinus tarsi was also examined by ultrasonography in a different scan plane; however, no saved image or video was available for presentation. Gait observation showed early heel-off during the right stance phase, with increased hip and knee flexion. Walking pain was 5/10 on the NRS.

Evaluation items included walking pain, PPT at the sinus tarsi, tactile hypersensitivity, ankle range of motion, ultrasonographic findings, and plantar pressure distribution during gait. Pain was scored using the NRS. Tactile hypersensitivity was assessed qualitatively as a change in sensation elicited by light brushing. Pressure pain threshold (PPT) was measured at the sinus tarsi using a Wagner algometer (Wagner Instruments, Greenwich, CT, USA). Ankle range of motion was measured with a goniometer for dorsiflexion with the knee flexed, dorsiflexion with the knee extended, and plantarflexion. Ultrasonography was performed using a LOGIQ P9 system (GE HealthCare, Chicago, IL, USA) to examine the anterior talofibular ligament, calcaneofibular ligament, and sinus tarsi. Gait analysis was performed using a zebris FDM-T treadmill system (WIN-FDM software, zebris Medical GmbH, Isny, Germany) at 2.0, 3.0, and 4.0 km/h, recording plantar pressure distribution and spatiotemporal parameters. As representative gait indices, this report presents the peak affected-side forefoot load (% body weight) and the center-of-pressure trajectory at 3.0 km/h, with forefoot loading compared with the unaffected side.

Taping intervention

Initial clinical reasoning considered that increased foot mobility after surgery had increased mechanical stress on the lateral ankle support structures, contributing to persistent pain. Taping (KT TAPE PRO, KT Health, American Fork, UT, USA) was therefore applied during daytime activity for four weeks to control lateral sway and reduce ligamentous stress. Although lateral sway appeared to decrease on observation, walking pain remained at NRS 5/10, with no sustained improvement. No clear change was observed on orthopedic testing, ultrasonographic evaluation, or ankle range of motion.

Reassessment

On reassessment, the pain site was more clearly localized to the sinus tarsi. Tactile hypersensitivity was noted at the sinus tarsi, and PPT was markedly reduced on the affected side (1.60 kgf) compared with the unaffected side (5.43 kgf). These findings suggested that mechanical stress from lateral ligament instability alone could not fully explain the pain, and that local pain hypersensitivity at the sinus tarsi might be involved. PPT had not been measured at the initial assessment; this reassessment represented the first measurement.

HVPC intervention

For pain hypersensitivity at the sinus tarsi, HVPC was applied using a Physio Active HV device (model PHV-1, Sakai Medical Co., Ltd., Tokyo, Japan). This device delivers a twin-peak pulsed current waveform with selectable polarity and an output frequency range of 1-200 Hz, pulse width range of 10-50 μsec, maximum output voltage of 300 Vp-p, and maximum output current of 40 mA rms at a 500-Ω load. In this case, the built-in preset Program 1 (“Pain”) was used, based on the manufacturer’s clinical application example for sinus tarsi syndrome. According to the manufacturer’s program information, Program 1 operates in a constant-frequency mode, with a fixed frequency of 200 Hz, a device-set pulse width of 20 μsec, and negative polarity. The device has no duty-cycle setting for this program, and stimulation was delivered continuously during each session.

**Figure 2:**
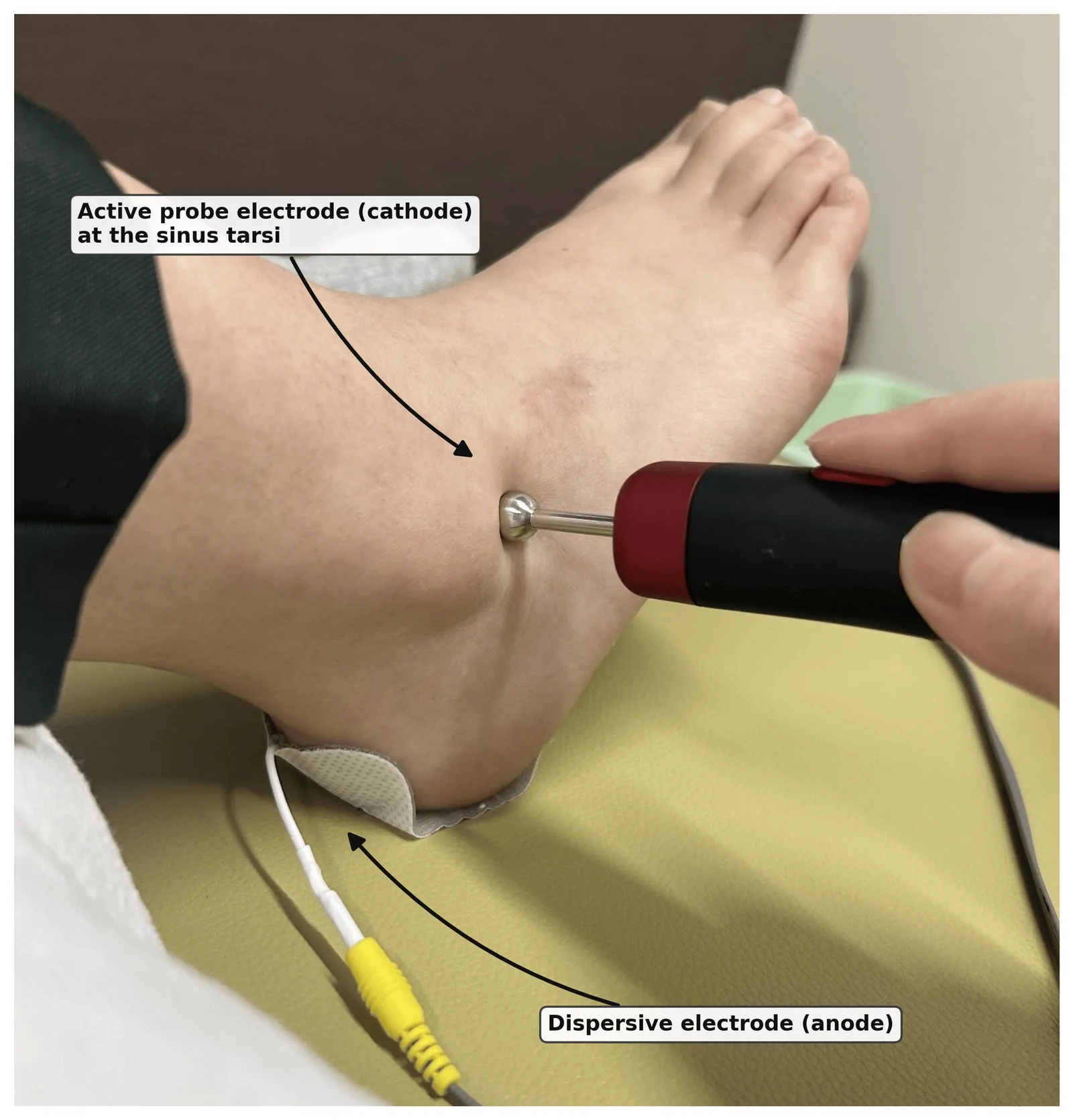
Electrode placement for HVPC Application of high-voltage pulsed current (HVPC). The active probe electrode (cathode) was applied to the sinus tarsi, and the dispersive electrode (anode) was placed over the calcaneus. The probe contact angle was measured at each session to standardize the stimulation site. Patient identifiers were removed.

A shooting probe with a 1-cm-diameter ball-tip electrode, straight type, was used. Because the sinus tarsi is a concave structure that is difficult to contact firmly with an adhesive pad electrode, the ball-tip probe electrode was selected to achieve stable contact with the target site. The active probe electrode (cathode) was applied to the sinus tarsi, and the dispersive electrode (anode) was placed over the calcaneus (Figure 2). The manufacturer’s clinical material describes an application example for sinus tarsi syndrome using Program 1 with the dispersive electrode placed over the calcaneus. In this case, the weight-bearing stimulation and concurrent ankle movement described in that material were not performed; instead, stimulation was applied at rest in a non-weight-bearing supine position. Stimulation intensity was set to the maximum the patient could tolerate, adjusted within a device-indicated range of 50-80 V. The displayed voltage value represented the effective output value from the device and did not represent the actual electrical current delivered into the body. Because the contact angle of the probe electrode can cause the stimulation site to shift, the contact angle was measured at each session to ensure that the same site and angle were targeted, thereby standardizing the procedure. Skin sensation was monitored throughout, and no adverse events occurred during or after any session.

Clinical course

**Figure 3:**
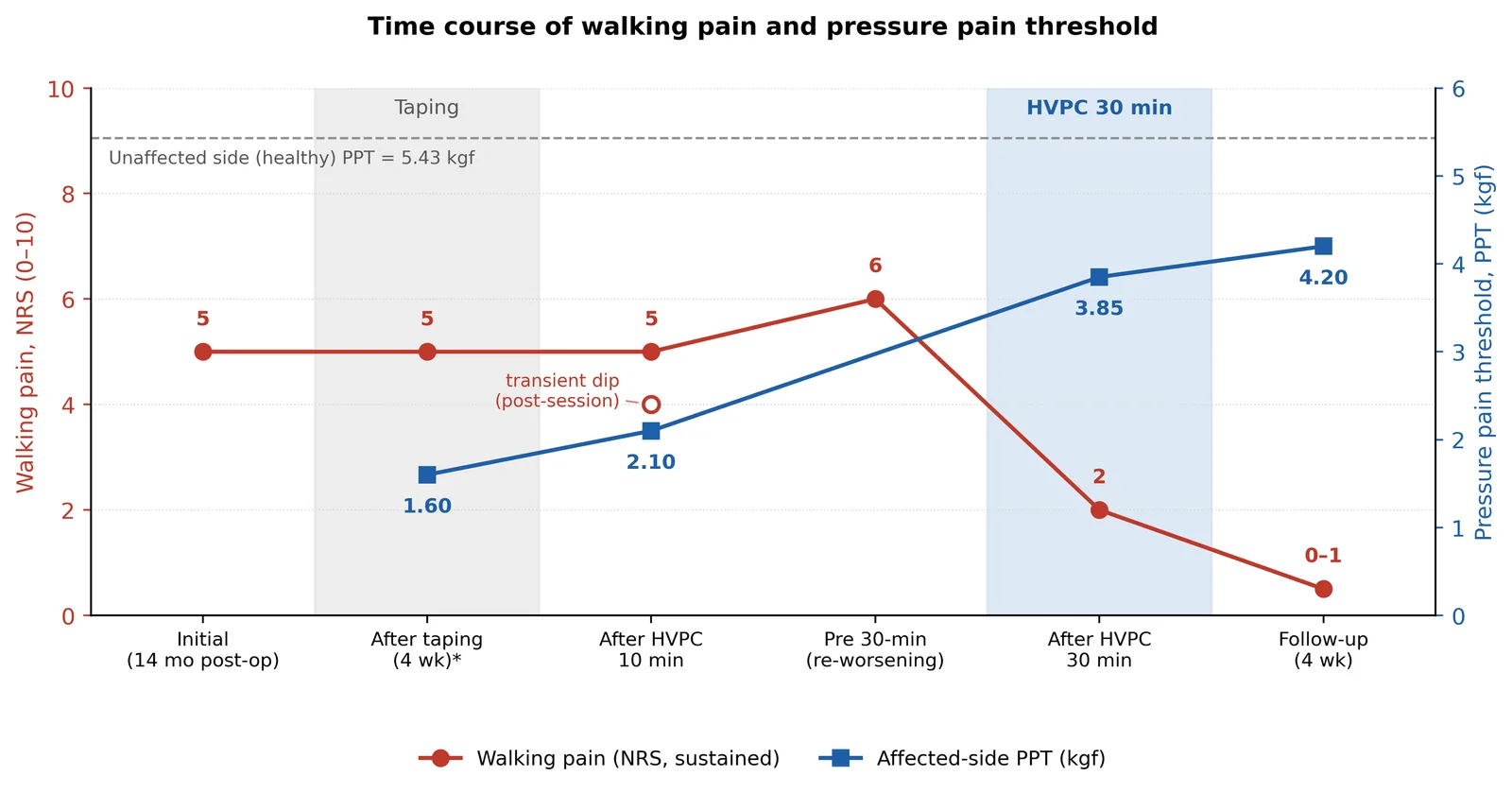
Time course of walking pain (NRS) and affected-side pressure pain threshold (PPT) Time course of sustained walking pain (NRS) and affected-side pressure pain threshold (PPT) at the sinus tarsi across the intervention phases. The open circle indicates the transient post-session decrease during the 10-minute block, during which sustained walking pain was unchanged; sustained pain re-worsened to 6/10 before the 30-minute block. The dashed line indicates the unaffected-side PPT for reference. *PPT was first measured at this timepoint (after the 4-week taping period); it was not measured at the initial evaluation. HVPC: high-voltage pulsed current; NRS: numerical rating scale

HVPC was first applied for 10 minutes per session, 4-5 times weekly for 5 weeks (22 sessions in total). Walking pain sometimes decreased transiently to NRS 4/10 immediately after a session, but sustained walking pain remained at NRS 5/10. It subsequently worsened to NRS 6/10 before the 30-minute protocol began. Meanwhile, PPT increased to 2.10 kgf on the affected side, although tactile hypersensitivity at the sinus tarsi persisted (Figure 3).

**Figure 4:**
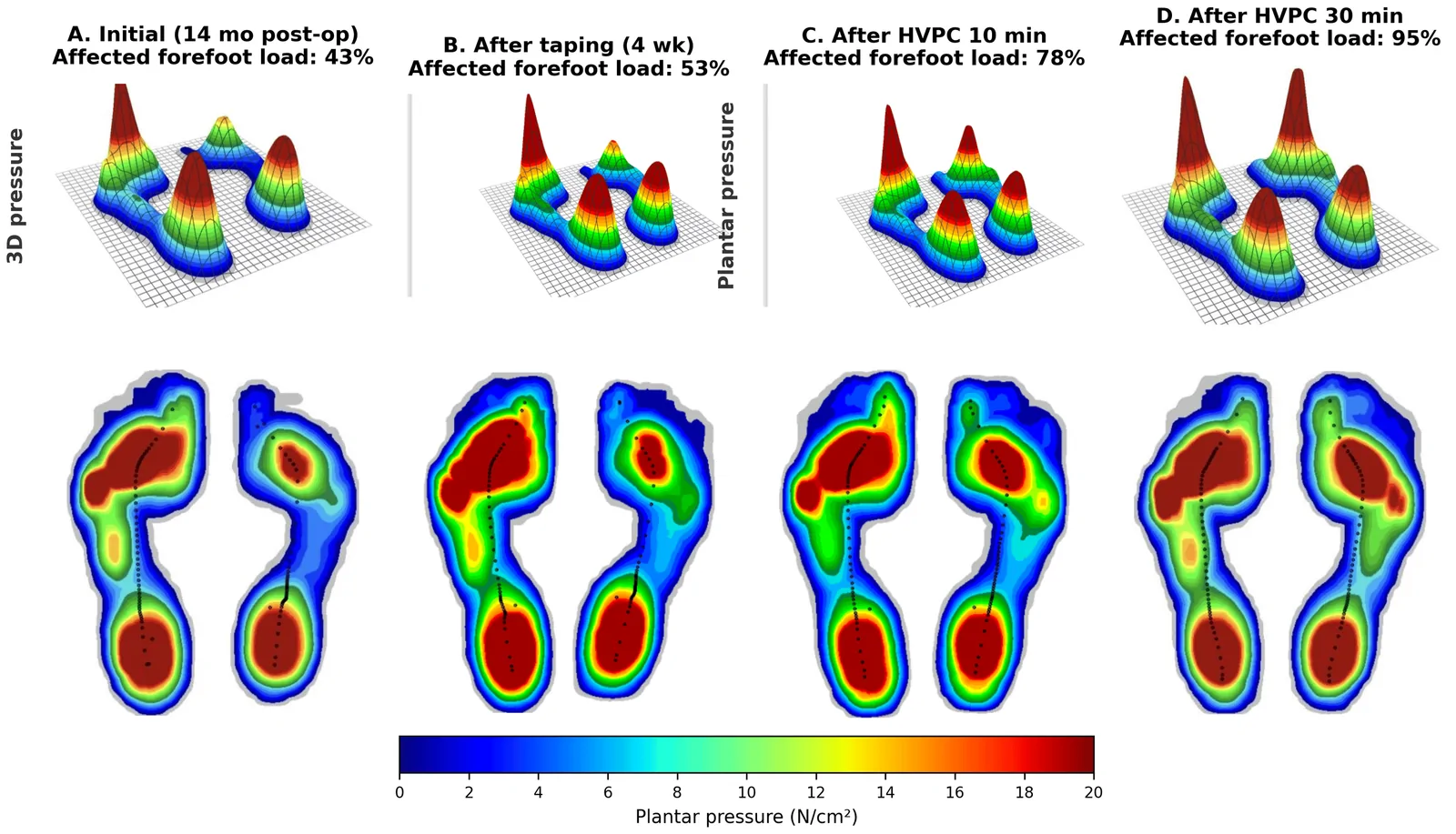
Plantar pressure distribution during gait Plantar pressure distribution during gait (3.0 km/h), computed as the cumulative distribution over a fixed 30-second period, at (A) initial evaluation, (B) after 4 weeks of taping, (C) after 10-minute high-voltage pulsed current (HVPC), and (D) after 30-minute HVPC. The top row shows the 3D pressure surface and the bottom row the corresponding 2D plantar pressure map. The affected forefoot peak load increased from 43% (A) to 95% (D) of body weight, approaching the unaffected side (~93-96%). Patient identifiers were removed.

Because the analgesic effect of 10-minute stimulation was not sustained, the stimulation duration was extended to 30 minutes per session while electrode placement and intensity range were kept unchanged. The 30-minute protocol was applied five times over one week. No physical therapy interventions other than taping and HVPC were performed throughout the observation period, and the type, dose, and frequency of analgesic medication remained unchanged. After the change to 30-minute stimulation, walking pain decreased to NRS 2/10, and PPT at the sinus tarsi increased to 3.85 kgf. On gait observation, the early heel-off seen before the intervention was delayed, and hip and knee flexion during stance decreased. Peak affected forefoot loading increased from 43% at initial evaluation, to 53% after taping, to 78% after 10-minute stimulation, and to 95% after 30-minute stimulation, approaching the unaffected side (approximately 93-96%) (Figure 4).

At the 4-week follow-up after completion of the 30-minute protocol, walking pain remained at NRS 0-1/10, PPT was 4.20 kgf, and peak affected forefoot loading was maintained at 92% (Table 1).

**Table 1:** Clinical course and outcomes NRS: numerical rating scale; PPT: pressure pain threshold; HVPC: high-voltage pulsed current (A)-(D) correspond to the panels in Figure 4. Peak forefoot load is the affected-side (right) peak forefoot load (% body weight); the unaffected side was approximately 93-96%.

Timepoint	Main intervention/assessment	Walking pain (NRS)	Affected PPT (kgf)	Gait/plantar pressure findings (peak affected forefoot load, %BW)
(A) Initial evaluation (14 mo post-op)	Physical therapy evaluation	5/10	Not measured	Early heel-off; increased hip/knee flexion; forefoot load 43%
(B) After taping (4 wk)	Daytime lateral-sway control; pain localized to sinus tarsi	5/10	1.60 (unaffected 5.43)	Lateral sway reduced but pain persisted; forefoot load 53%
(C) After 10-min HVPC	10 min/session, 4–5×/wk, 5 wk, 22 sessions total	Sustained 5/10 (transient 4/10 post-session)	2.10	Sustained gait improvement inadequate; forefoot load 78%
Before 30-min block (re-worsening)	Observation after 10-min protocol	6/10	—	Pain re-worsened
(D) After 30-min HVPC	30 min/session, 5× over 1 wk	2/10	3.85	Delayed heel-off, reduced hip/knee flexion; forefoot load 95%
Follow-up (4 wk after 30-min protocol)	Observation	0–1/10	4.20	Forefoot load 92%, no adverse events

## Discussion

Clinical changes after extension of stimulation duration

The 10-minute HVPC protocol produced only transient pain relief, without sustained improvement in walking pain. After the stimulation duration was sequentially extended to 30 minutes while maintaining the same electrode placement and intensity range, improvements were observed in NRS, PPT, gait observation, and plantar pressure distribution. These changes, however, should be interpreted as a temporal association rather than evidence of a causal effect of the longer stimulation duration. Multiple mechanisms have been proposed for the analgesic effect of electrical stimulation, including inhibition of nociceptive input at the dorsal horn and activation of descending pain-inhibitory pathways [8]. The Program 1 (“Pain”) setting and electrode placement used in this case were based on the manufacturer’s clinical application example for sinus tarsi syndrome; however, the weight-bearing stimulation and concurrent ankle movement described in that material were not performed, and stimulation was instead applied at rest in a non-weight-bearing position. Because the sinus tarsi is a concave structure, the ball-tip probe electrode may have helped stabilize contact with the target region. Nevertheless, the mechanism underlying the observed clinical changes was not directly examined.

No physical therapy interventions other than taping and HVPC were performed throughout the observation period, and analgesic medication type, dose, and frequency remained unchanged. The improvements in NRS, PPT, gait observation, and plantar pressure distribution observed after the change to 30-minute stimulation therefore appeared to be temporally associated with the change in intervention conditions. However, because this was a sequential single-case design without a control condition, washout period, or repeated baseline, alternative explanations cannot be excluded. These include cumulative treatment effects from the preceding 10-minute protocol, natural recovery, placebo response, regression to the mean, changes in activity level, and expectation effects. Therefore, the observed improvement cannot be attributed solely to the extended stimulation duration, and this finding should be interpreted as a hypothesis-generating observation.

Clinical implications

This case suggests that in persistent postoperative lateral ankle pain after tarsal coalition resection, the pain site and underlying mechanism should be repeatedly reassessed. In particular, when mechanical control alone does not achieve adequate improvement, evaluating tenderness, sensory hypersensitivity, and PPT at the sinus tarsi to consider the contribution of localized mechanical pain hypersensitivity may be useful. When applying electrical stimulation therapy, it is also important to adjust parameters such as stimulation duration and intensity according to the patient’s response, rather than simply deciding whether to apply the modality.

Limitations

This report has several limitations. First, it describes a single case, and the observed clinical course cannot be generalized. Second, the comparison between the 10-minute and 30-minute protocols reflects a sequential change over time, without a concurrent control condition, washout period, or repeated baseline. Third, localized mechanical pain hypersensitivity was inferred from clinical findings and PPT, without neurophysiological verification or standardized quantitative sensory testing. Fourth, although changes in NRS, PPT, gait observation, and plantar pressure distribution were clinically notable within this case, the minimal clinically important difference for these combined outcomes has not been established for persistent postoperative lateral ankle pain after talonavicular coalition resection. Therefore, the magnitude of change should be interpreted cautiously. Fifth, PPT and gait assessments were used as supportive clinical outcome measures, but measurement variability and the absence of repeated baseline measurements limit the strength of interpretation. Sixth, detailed three-dimensional gait analysis, including joint angles and lower-limb segmental motion, was not performed. Seventh, although medication use remained constant, a pharmacological contribution to pain relief cannot be entirely excluded. Eighth, although the preset frequency, device-set pulse width, polarity, waveform characteristics, and duty-cycle status were confirmed using the manufacturer’s program information, the actual electrical current delivered into the body was not measured in this case. Future work should include studies involving multiple cases and optimization of HVPC parameters, including stimulation duration, frequency, intensity, and electrode configuration.

## Conclusions

This case describes a patient with persistent lateral ankle pain after talonavicular coalition resection in whom both mechanical factors and localized mechanical pain hypersensitivity at the sinus tarsi were considered during repeated clinical reasoning. Taping aimed at reducing mechanical stress did not produce sustained pain relief. Clinical improvements in walking pain, PPT, gait findings, and plantar pressure distribution were observed after sequential extension of HVPC stimulation duration from 10 to 30 minutes while maintaining the same electrode placement and intensity range. However, because this was a sequential single-case report without a control condition, washout period, or repeated baseline, these findings should be interpreted as a temporal association rather than evidence of a causal effect of the 30-minute HVPC protocol. This case provides a hypothesis-generating observation that stimulation duration may be a clinically relevant parameter when adjusting electrical stimulation based on patient response, but further studies are needed to determine causality and generalizability.
